# The human epidermal growth factor receptor (EGFR) gene in European patients with advanced colorectal cancer harbors infrequent mutations in its tyrosine kinase domain

**DOI:** 10.1186/1471-2350-12-144

**Published:** 2011-10-25

**Authors:** Brigitte Metzger, Laetitia Chambeau, Dominique Y Begon, Carlo Faber, Jacques Kayser, Guy Berchem, Marc Pauly, Jacques Boniver, Philippe Delvenne, Mario Dicato, Thomas Wenner

**Affiliations:** 1 Laboratoire de Recherche sur le Cancer et les Maladies du Sang, 4, rue Ernest Barblé L-1210 Luxembourg, Luxembourg; 2 Département d'anatomie pathologique, Université de Liège, Avenue de l'Hôpital, 1 (B34), GIGA-Research, CHU Sart-Tilman, 4000 Liège, Belgium; 3 Service de Chirurgie, Clinique Ste Thérèse, ZithaKlinik, 36, rue Sainte Zithe, L-2763 Luxembourg, L-2763 Luxembourg; 4 Service d'Hémato-Cancérologie, Centre Hospitalier, 4, rue Ernest Barblé L-1210 Luxembourg Luxembourg

## Abstract

**Background:**

The epidermal growth factor receptor (EGFR), a member of the ErbB family of receptors, is a transmembrane tyrosine kinase (TK) activated by the binding of extracellular ligands of the EGF-family and involved in triggering the MAPK signaling pathway, which leads to cell proliferation. Mutations in the EGFR tyrosine kinase domain are frequent in non-small-cell lung cancer (NSCLC). However, to date, only very few, mainly non-European, studies have reported rare EGFR mutations in colorectal cancer (CRC).

**Methods:**

We screened 236 clinical tumor samples from European patients with advanced CRC by direct DNA sequencing to detect potential, as yet unknown mutations, in the *EGFR *gene exons 18 to 21, mainly covering the EGFR TK catalytic domain.

**Results:**

EGFR sequences showed somatic missense mutations in exons 18 and 20 at a frequency of 2.1% and 0.4% respectively. Somatic SNPs were also found in exons 20 and 21 at a frequency of about 3.1% and 0.4% respectively. Of these mutations, four have not yet been described elsewhere.

**Conclusions:**

These mutation frequencies are higher than in a similarly sized population characterized by Barber and colleagues, but still too low to account for a major role played by the *EGFR *gene in CRC.

## Background

The epidermal growth factor receptor (EGFR) is a cell surface receptor belonging to the ErbB family of receptors, a family of four tyrosine kinase receptors, EGFR (ErbB-1), HER2/c-neu (ErbB-2), Her3 (ErbB-3) and Her4 (ErbB-4). These tyrosine kinases are involved in various aspects of cell growth and survival and have been implicated in the initiation and progression of several types of human malignancies. EGFR is involved in cell growth control through its role in the mitogen-activating-protein-kinase (MAPK) pathway. Therefore, over-expression or mutation of EGFR may be responsible for the constitutive activation of the pathway which these receptors control[1]. In colorectal cancer (CRC), the *EGFR *gene has been found to be over expressed in more than 80% of tumors and is significantly associated with TNM stage T3[2]. Hence, EGFR might be involved in the development of colorectal cancer and has now been validated as a clinically relevant target. At present, two different monoclonal antibodies raised against the extracellular part of the EGFR receptor are in use in a clinical setting (Cetuximab and Panitumumab). Both of these monoclonal antibodies bind to the extracellular domain of the EGFR preventing its activation but, Cetuximab and Panitumumab have single-agent response rates in the region of only 10%[3,4]. K-ras mutational status could in part explain this poor outcome as mutation in the K-ras gene would activate the EGFR pathway whatever the activation of EGFR receptor is activated or blocked. However 40% to 70% of patients with a wild type K-ras won't benefit from the use of these monoclonal antibodies[5-7]. This might be explained by the occurrence of mutation in the same pathway downstream of K-ras, as B-raf for example, or by the variation of the EGFR receptor copy number[8-10]. This might also be explained by the occurrence of mutations in the *EGFR *gene as shown in non-small-cell lung cancer (NSCLC), where mutations in exons 18-21 coding for the tyrosine kinase domain are frequent (10-50%)[11]. These mutations, however, are generally related to an increased sensitivity to EGFR inhibitors, which might not be the case in CRC.

There are only a few reports of mutations in the *EGFR *gene in colorectal tumors and all of these used sequencing after gene amplification. The first report focused on only 31 cases from Milan (Italy) and is the only European study[12]. Of these 31 cases, only one showed a mutation. The second study focused on 33 cases and was performed on Japanese patients from Beppu[13]. Of these 33 cases, ten were detected as having mutations in the *EGFR *gene. The third focused on 101 Chinese (Shangaï) patients and the authors described only 2 mutations[14]. The last study was conducted by Barber *et al*.,[15] in Baltimore USA, where only one mutation in 293 patients was characterized.

Since *EGFR *mutations in NSCLC have a substantially higher frequency in East Asians than in other ethnicities, a relationship between *EGFR *mutation frequency and ethnicity has been established[16]. We studied a large cohort of a European population in order to investigate the frequency of occurrence of mutations in the *EGFR *gene in a European context and to determine if these mutation occur as frequently as in the American population. We also determined the somatic nature of the mutation by using the corresponding patient's blood DNA as the control.

## Methods

### Patients

Two hundred and thirty-six tumor samples were collected between 2002 and 2007, were obtained from patients with CRC with the agreement of the Luxembourg Ethics Committee and informed consent were obtained from all participants. All patients were of European ethnicity. The samples had a histological stage equal to or greater than pT1.

### Clinical tumor samples and non-tumor control samples

Colorectal tumor samples were isolated during surgical resection and, in order to obtain more than 80% of the cancer tissue, a small part of the middle of the tumor was rapidly frozen by the clinician in liquid nitrogen and stored at -80°C until genomic DNA preparation. As a non-tumor tissue control, whole leukocyte samples were collected from the same patients in vacutainers/EDTA (Becton Dickinson, Rutherford, New Jersey, USA) and extraction was carried out using NH_4_Cl treatment. These control cells were used to determine whether these mutations were germline or somatic for each mutation found (i.e. silent or missense). Hence, each time we found a mutation in an exon, the corresponding leukocyte DNA was sequenced in both directions in the same exon.

### Genomic DNA preparation

Cellular genomic DNA was extracted from roughly 25 mg tumor tissue or non-tumor leukocyte control samples using the DNeasy Blood & Tissue kit (Qiagen, Westburg, Leusden, the Netherlands) and DNA concentrations were checked using a nanodrop ND1000 apparatus (ThermoFisher, Aalst, Belgium) according to the manufacturer's instructions.

### PCR amplification

Specific PCR primers (Eurogentec, Seraing, Belgium) for the amplification of the DNA regions covering exons 18, 19, 20 and 21, respectively, of the human EGFR gene tyrosine kinase domain were designed according to [1] (Table 1).

**Table 1:** Sequences of primers used to amplify and sequence EGFR exons 18 to 21

Exon	Sequence 5'→3'	Tm (°C)
18	for CAAATGAGCTGGCAAGTGCCGTGTC rev GAGTTTCCCAAACACTCAGTGAAAC	58

19	for GCAATATCAGCCTTAGGTGCGGCTC rev CATAGAAAGTGAACATTTAGGATGTG	67

20	for CCATGAGTACGTATTTTGAAACTC rev CATATCCCCATGGCAAACTCTTGC	58

21	for CTAACGTTCGCCAGCCATAAGTCC rev GCTGCGAGCTCACCCAGAATGTCTGG	67

The annealing temperature is shown. For and rev are primers forward and reverse respectively according to the gene transcription orientation. Forward primers were used to sequence all PCR products and reverse primers to confirm the presence of mutation.

PCR amplification was performed using the AmpliTaq Gold^® ^DNA polymerase (Applied Biosystems, Halle, Belgium) according to the manufacturer's protocol using 100 ng of genomic DNA in an Eppendorf Mastercycler (Eppendorf, Fisher Scientific, Aalst, Belgium). The PCR program was set up with a denaturizing step of 10 min at 95°C followed by 30 repetitions of the following sequence: 95°C, 1 min; annealing temperature according to the primer used (Table 1), 1 min; 72°C, 1 min; and a final elongation step of 10 min. The resulting PCR products were checked on 2% agarose gel electrophoresis and purified using the ExoSAP-IT^® ^purification method (USB Affymetrix, Cleveland, USA).

KRAS mutations located within codons 12 and 13 were characterized by direct sequencing according to the conditions published in [17].

### Direct DNA sequencing

Once purified, the PCR products were analyzed by direct automatic PCR-assisted DNA dideoxynucleotide sequencing according to the manufacturer's protocol using the oligonucleotides previously described (Big Dye Terminator V3.1, Applied Biosystems, Halle, Belgium). Analysis of the purified sequenced products was performed on a 3130 Genetic Analyzer (Applied Biosystems, Halle, Belgium). Where there was the possibility of a mutation, sequencing was repeated at least once in the antisense direction for confirmation as well as on a new PCR product and in the corresponding leukocyte control.

### DNA mutation analysis

DNA sequence data were examined for potential point mutations, deletions and insertions using the SeqScape software version 2.5 (Applied Biosystems, Halle, Belgium).

## Results

Cellular genomic DNA was extracted from clinical tumor tissue, adenoma-carcinomas, or from leukocytes as control samples, from 236 European CRC patients in Luxembourg. Single nucleotide polymorphisms (SNP's) and mutations were detected and analyzed using the EGFR gene card website, the cosmic database and the EGFR database.

DNA regions covering exons 18, 19, 20 and 21 of the human *EGFR *gene encompassing mainly the tyrosine kinase domain were then amplified using PCR, and directly sequenced. Missense mutations arose at a frequency of 2.1%, 0%, 0.4% and 0.4% in exons 18 to 21 respectively whereas these samples showed an overall frequency of mutation of 2.6%, 0.5%, 0.8%, and 3.8% in exons 18 to 21 respectively. Among these mutations, we were able to distinguish those already described from non described missense mutations (affecting protein sequence) and silent point mutations (mutations affecting DNA sequences without affecting protein sequence). Moreover, each time a mutation was characterized in an exon, it was confirmed by a reverse sequencing in the same PCR product and on a new one (both ways) and the same exon was sequenced in the blood DNA of the patient where this mutation was found (both ways).

Sometimes we ran out of colorectal tissues from some patients and were therefore unable to sequence 236 samples for all the four exons. We were, nevertheless, able to sequence 188, 221, 227 and 236 samples for exons 18, 19, 20 and 21, respectively.

**Figure 1:**
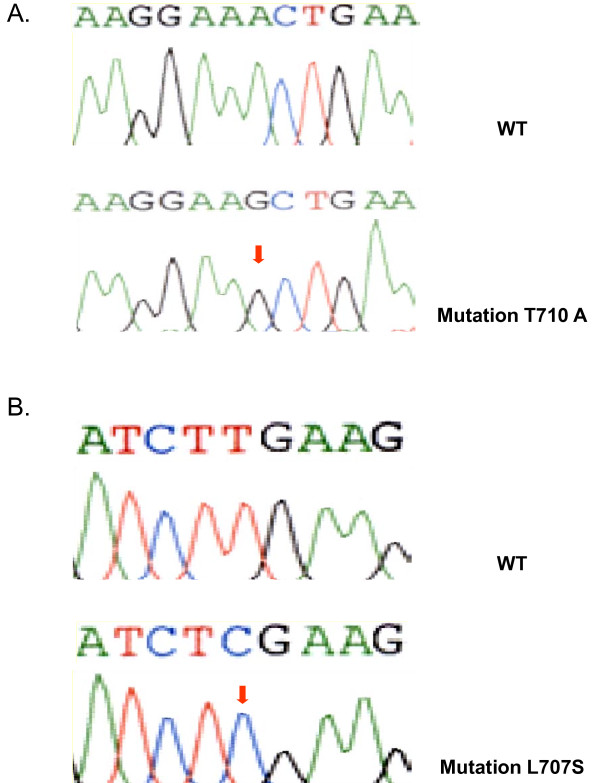
Comparison between wild type and mutant electropherograms in A and B showing two novel mutations in exon 18 of the EGFR gene.

In exon 18, missense point mutations were present with a frequency of only 2.1% (4 out of 188 cases determined (Figure 1 and Table 2). Only one mutation had already been described and this affected codon 712 (TTC to TCC transition, F712S). The three remaining mutations had not yet been described in CRC nor in other cancers according to the EGFR databases and these affected codons 707 (TTG to TCG transition, L707S), 710 (ACT to GCT transition, T710A) and 711 (GAA to GTT double transversion Q711V). The missense mutations were all somatic, since none was found in the non-tumor control tissue.

**Table 2:** Mutations found in EGFR exons 18 to 21

Exon	Frequency	Mutation type	Heredity	K-ras status
		**TTG to TCG (L707S) n2120 Ti**	s	wt
		**ACT to GCT (T710A) n2128 Ti**	s	wt
18	5/188 (2.6%)	**GAA to GTT (E711V) n2132/2133 Tv**	s	wt
		TTC to TCC (F712S) n2135 Ti	s	wt
		*ACG to ACA (T725T) n2175 Ti*	g	wt
19	1/221 (0.5%)	***GTC to GTT (V742V) n2226 Ti***	g	wt
		TTC to TCC (F795S) n2384 Ti	s	wt
20	2/227 (0.8%)	GGC to AGC (G796S) n2386 Ti	g	Wt
		*CGC to CGT (R836R) n2508 Ti*	g	M (13)
		*CGC to CGT (R836R) n2508 Ti*	g	wt
		*CGC to CGT (R836R) n2508 Ti*	g	wt
		*CGC to CGT (R836R) n2508 Ti*	g	wt
21	9/236 (3.8%)	*CGC to CGT (R836R) n2508 Ti*	g	wt
		*CGC to CGT (R836R) n2508 Ti*	g	wt
		*CGC to CGT (R836R) n2508 Ti*	s	M (12)
		*CGC to CGT (R836R) n2508 Ti*	nd	wt
		***ACA to AGA (T847R) n2540 Tv***	g	Wt

Frequency as well as mutation type and heredity are shown. Bold characters underline mutations not described elsewhere and italic characters indicate silent point mutations. Nucleotides positions are marked with an n followed by the position number. Transversion or transition mutation types are marked by Tv or Ti respectively. Germline and somatic mutations are designated by g and s respectively and correspond to mutations found (germline, g) or not found (somatic, s, tumor specific) in peripheral blood-DNA of the same patients. KRAS mutations located within the codon 12 (M 12) or 13 (M13) are highlighted.

The last mutation found in exon 18 was a silent germinal point mutation and corresponds to a SNP, at codon 725 (ACG to ACA transition, Y725Y) in one patient (Table 2). Nucleotide positions mutated are detailed in table 2.

In exon 19, only one mutation was detected out of 221 samples determined (frequency 0.5%, Table 2). This mutation has yet not been described and might be a new SNP since it consisted of another silent germline point mutation found at codon 742 (GTC to GTT transition, V742V).

In exon 20, two already described missense mutations out of 225 determined samples (frequency 0.8%) were characterized, one somatic mutation affecting codon 795 (TTC to TCC transition, F795S) and one germinal mutation in codon 796 (GGC to AGC transition, G796S). We also detected an already described SNP, silent point mutation in codon 787, leading to a G to A transition (CAG-CAA - Q787Q, unchanged), in 186 of the 225 tumors analyzed (frequency 82.7%, 11 undetermined). This SNP was either homozygous (93 cases, 41.3%) or heterozygous (G/A, 93 cases, 41.3%, Figure 1, Table 3). The SNP was considered heterozygous when an electropherogram showed a superimposition of the signals corresponding to G and A at roughly half the intensity of the one obtained in homozygous sequences. These silent point mutations were nearly all germinal as 91 heterozygous mutations and 92 homozygous mutations were found both in tumors and in patient leukocyte DNA (Table 3). There were only 3 cases where a wild type sequence was found in leukocytes and had mutated in tumors (2 heterozygous and 1 homozygous) and surprisingly, 4 patients showed a mutated *EGFR *in their leukocyte DNA but a wild type sequence in their tumors (2 heterozygous, 2 homozygous). In order to check whether this silent point mutation was more frequently found in patients suffering from the disease, we sequenced DNA from the leukocytes of healthy volunteers. A similar frequency of occurrence of this silent point mutation was found, as 56 out of 61 controls (91.8%) were shown to harbor this silent point mutation (34 heterozygotes and 22 homozygotes).

**Table 3:** Combination of wild type and mutated exon 20 sequence in codon 787 of the EGFR gene

WBC Tumor	CAG (WT)	CA G/A Polym' het'	CAA Polym' hom'	Total
CAG (WT)	25	2	2	39
CA G/A Polym' het'	2	91	0	93
CAA Polym' hom'	1	0	92	93

Total	38	93	94	225

Wild type (WT) and mutant (polymorphic heterozygous (polym het) or homozygous (polym hom)) sequences of EGFR exon 20 codon 787 have been found in tumors and peripheral blood-DNA (WBC: White Blood Cells).

Finally, we characterized a still non described missense mutation at codon 847 (ACA to AGA transversion, T847R, frequency 1 out of 236 (0.4%) in exon 21. This mutation was germinal as the same mutation was found in the leukocytes of this patient. Moreover, an already described silent SNP was found in 8 patients out of 236 tumors analyzed at codon 836 (CGC to CGT transition, R836R). Six of these mutations were germline and one somatic, the remaining mutation was undetermined (not sequenced).

## Discussion

To summarize, missense mutations arose at a frequency of 2.1%, 0%, 0.4% and 0.4% in exons 18 to 21 respectively whereas these samples showed an overall frequency of mutation of 2.6%, 0.5%, 0.8%, and 3.8% in exons 18 to 21 respectively. This included silent point mutation except for exon 20 where a frequent silent point mutation (82.7%) was characterized. These frequencies are within the range of those reported elsewhere, in terms of over-all frequency of missense mutation occurrence in exons 18 to 21, 0.34 to 12% [10-13,15,18-20]. Hence, our cohort may be representative of the disease.

Unlike in NSCLC, where such mutations occur at a frequency of up to 45%[1,21], neither small in-frame deletions, double mutations (silent and missense) nor insertions were found. In addition, in the case of missense mutations, transitions (4 somatic 1 germline, see Table 2) were far more frequent (71.43%, 5 out of 7) than transversions. Moreover, 71.43% of these mutations were somatic and therefore specific to the tumor whereas the remaining mutations were germline, since they were also found outside the tumor, namely in the normal control tissue represented by leucocytes from the same patient. Most of these germline mutations were already known SNPs except in exon 19 (V742V) which has not yet been described and might be a new SNP, and in exon 20 (G796S) which might also be a new SNP although this mutation has been described in prostate cancer as tumor specific[22]. Most of these mutations were located in exon 18 corresponding to the ATP nucleotide binding loop of the EGFR tyrosine kinase domain (codons 707, 710, 711 and 712, 57.14%, 4 out of 7), two were located in exon 20 (codons 795 and 796) and one in exon 21 at codon 847, corresponding to the kinase activity loop. None was found in exon 19.

At least one silent point mutation was observed in each exon (codons 725, 742, 787 and 836). These were all germline, except in one tumor at codon 836, where the mutation was not found in the leukocytes from the patient.

Moreover, in this study, we found an already known silent SNP in *EGFR *exon 20 (Q787Q) arising at a very high frequency (186 out of 225 analyzed, 82.7%). This silent mutation has already been described in several cases of NSCL or head and neck squamous carcinoma[23,24] but only once in CRC,[13] occurring less frequently.

Our main objective was to check if EGFR mutation frequency might explain the poor efficacy of the use of monoclonal antibody raised against the extracellular part of the EGFR receptor. As this may be partially explained by the occurrence of mutation in K-ras gene which led to the constitutive activation of the K-ras pathway, downstream of the EGFR receptor, we tested the K-ras mutational status of our patient harboring a mutation in the EGFR gene (Table 2). The K-ras gene has been found mutated in two cases (2 out of 17 cases, 12%), when EGFR showed a SNP in codon 21. The small number of somatic missense mutation (5 cases) found in our study doesn't allow us to get statistical significant results but we can underline that none of these patients showed mutations in K-ras whereas K-ras mutation frequency in colorectal cancer vary from 30% to 40%.

Moreover, the comparison of the mutational status and clinical parameters which would have been of great interest is not possible due to the small number of somatic missense mutation (5 cases) found in our study. A larger cohort would be needed to address these questions.

Functional analysis would also be of great interest to study the impact of the new mutations described in this work on EGFR expression and signaling.

## Conclusions

In conclusion, missense mutation, which may be responsible for an activation of the *EGFR *gene through amino acid substitution, seems not to play a major role in the occurrence and development of colorectal cancer. However, the frequency of mutation in the population in our study is much higher than in a similarly sized population characterized by Barber and colleagues,[15] and four new EGFR mutations were described.

## Competing interests

The authors declare that they have no competing interests.

## Authors' contributions

BM, LC, DB, MP performed research, CF and JK collected biologic samples, MD, JB, PD coordinated the research, GB designed and coordinated the research, TW designed, coordinated and performed research, interpreted data, and wrote the article. All authors read and approved the final manuscript.

The EGFR databases:

http://www.egfr.org

http://www.sanger.ac.uk/perl/genetics/CGP/cosmic

http://www.genecards.org/

## Pre-publication history

The pre-publication history for this paper can be accessed here:

http://www.biomedcentral.com/1471-2350/12/144/prepub
